# VCIP135 associates with both the N- and C-terminal regions of p97 ATPase

**DOI:** 10.1016/j.jbc.2023.105540

**Published:** 2023-12-10

**Authors:** Suzune Nakayama, Hisao Kondo

**Affiliations:** Department of Molecular Cell Biology, Graduate School of Medical Sciences, Kyushu University, Fukuoka, Japan

**Keywords:** Golgi, endoplasmic reticulum (ER), membrane fusion, ATPases associated with diverse cellular activities (AAA), SNARE, complex

## Abstract

Two distinct p97ATPase-mediated membrane fusion pathways are required for Golgi and endoplasmic reticulum (ER) biogenesis, namely, the p97/p47 pathway and the p97/p37 pathway. p97 (VCP)/p47 complex-interacting protein p135 (VCIP135) is necessary for both of these pathways. Although VCIP135 is known to form a complex with p97 in the cytosol, the role of this complex in Golgi and ER biogenesis has remained unclear. In this study, we demonstrated that VCIP135 has two distinct p97-binding sites at its N- and C-terminal regions. In particular, the C-terminal binding site includes the SHP motif, which is also found in other p97-binding proteins, such as p47, p37, and Ufd1. We also clarified that VCIP135 binds to both the N- and C-terminal regions of p97; that is, the N- and C-terminal binding sites in VCIP135 interact with the C- and N-terminal regions of p97, respectively. These two interactions within the complex are synchronously controlled by the nucleotide state of p97. We next generated VCIP135 mutants lacking each of the p97-binding sites to investigate their functions in living cells and clarified that VCIP135 is involved in Golgi and ER biogenesis through its two distinct interactions with p97. VCIP135 is hence a unique p97-binding protein that functions by interacting with both the N-and C-terminal regions of p97, which strongly suggests that it plays crucial roles in p97-mediated events.

The endoplasmic reticulum (ER) and Golgi apparatus play central roles in intracellular membrane traffic. Newly synthesized secretory and membrane proteins are incorporated into the ER, and then transported to the Golgi during which they receive various post-translational modifications. The Golgi apparatus dispatches all of these proteins toward their correct downstream locations (1). Closely associated with the central roles of the ER and Golgi are their unique and complex architectures; that is, the ER has a network structure and the Golgi consists of a series of stacked flattened cisternae (2). How such complicated structures of the ER and Golgi are built and maintained is one of the most important issues in molecular cell biology.

We previously reported that p97ATPase is required for Golgi biogenesis, together with its cofactors, p47 and p37. Specifically, p97ATPase and its cofactor are thought to function in soluble NSF attachment protein receptor (SNARE) priming (3, 4).

p97 forms a complex with p47, and the complex induces Golgi membrane fusion (3). As p47, which contains nuclear localization signals in its peptide sequence, mainly localizes in the nucleus during interphase, the p97/p47 pathway is thought to be specifically involved in the reassembly of organelles at the end of mitosis (5). Recently, we also showed that p97 binds to formiminotransferase cyclodeaminase (FTCD) to form the tethering complex FTCD-p97-p47-FTCD during the p97/p47-mediated Golgi reassembly at the end of mitosis (6). The p97-p47 complex is therefore thought to play dual roles of membrane tethering and SNARE priming in the membrane fusion process.

In contrast, p37, which is another cofactor of p97, localizes to the cytoplasm during interphase and also induces Golgi membrane fusion together with p97 (4). The p97/p37 pathway is hence thought to be required for organelle maintenance during interphase. The p97/p37 pathway, as well as the NSF pathway, utilizes p115-GM130 tethering for Golgi membrane fusion (4).

The group of Schekman originally demonstrated that ER membrane fusion is mediated by Cdc48p, the yeast orthologue of p97, using an *in vitro* system (7). We also reported the requirement of p97, p47, and p37 for ER network formation using both *in vitro* and *in vivo* systems (4, 8, 9). Based on these findings, both the p97/p47 and p97/p37 pathways are thought to function in ER biogenesis, although their roles in the ER still remain unclear.

Both p97 pathways require p97 (VCP)/p47 complex-interacting protein, p135 (VCIP135) for their Golgi and ER membrane fusion activities (4, 8, 9). VCIP135 is a cytosolic protein and was originally identified as a p97/p47 complex-interacting protein, which binds to the p97-p47 complex and assists its dissociation *via* p97-catalyzed ATP hydrolysis (8). Wang *et al.* reported that VCIP135 has deubiquitinating activity, which is essential for p97/p47-mediated Golgi membrane fusion (10, 11). The deubiquitinating activity of VCIP135 is, however, unnecessary for p97/p37-mediated Golgi membrane fusion, although VCIP135 itself is essential in this pathway (4). In ER membrane fusion, neither the p97/p47 pathway nor the p97/p37 pathway requires the deubiquitinating activity of VCIP135 (9). Therefore, it is likely that VCIP135 works in two distinct ways; *i.e.*, *via* its deubiquitinating activity, and in a ubiquitin-independent manner. On the other hand, VCIP135 is known to bind to p97 in the cytosol (8). However, VCIP135 was reported to function without the assistance of p97 in the activation of SPRTN, a specialized DNA-dependent metalloprotease (12). Additionally, the binding of VCIP135 to p97 was reported to cause no change in its deubiquitinating activity (9). To date, there are no lines of evidence indicating that VCIP135 requires its binding to p97 for Golgi and ER biogenesis.

In this study, we investigated the binding interactions between VCIP135 and p97 and found that VCIP135 has two distinct p97-binding sites; that is, its N- and C-terminal p97-binding sites interact with the C- and N-terminal regions of p97, respectively. We next generated VCIP135 mutants lacking each of the p97-binding sites, and by expressing these mutants in living cells, clarified that VCIP135 plays roles in Golgi and ER biogenesis through its two distinct interactions with p97. These results indicate that VCIP135 is a unique p97-binding protein that functions by interacting with both the N- and C-terminal regions of p97.

## Results

### VCIP135 has two p97-binding sites

**Figure 1:**
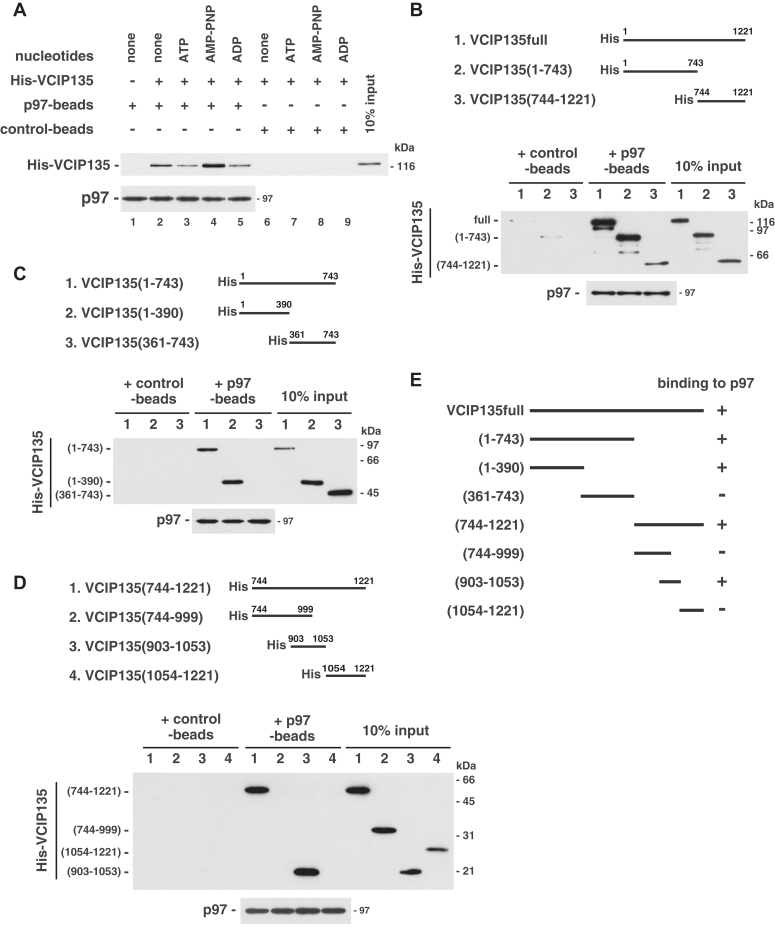
**VCIP135 has two p97-binding sites.***A*, VCIP135 binds to p97 in a nucleotide-dependent manner. Endogenous p97 was biotinylated and immobilized onto streptavidin beads. The p97-immobilized beads (p97-beads; p97, 0.40 μg) were incubated with His-tagged VCIP135 (1.0 μg) in the presence of the indicated nucleotide (1 mM), and the bound proteins were fractionated by SDS-PAGE, followed by Western blotting with antibodies to p97 and His-tag. *B*, both the N- and the C-terminal halves of VCIP135 bind to p97. p97-immobilized beads (p97-beads; p97, 0.75 μg) were incubated with either His-tagged full-length VCIP135 (1.2 μg), its N-terminal half [(1-743), 1.1 μg] or C-terminal half [(744-1221), 0.60 μg] in the presence of 1 mM AMP-PNP. The blots were probed with antibodies to p97 and His-tag. *C*, the p97-binding site in the N-terminal half of VCIP135. p97-immobilized beads (p97-beads; p97, 0.50 μg) were incubated with either the N-terminal half of VCIP135 or its fragments [(1-743), 1.1 μg; (1-390), 0.67 μg; (361-743), 0.77 μg] in the presence of 1 mM AMP-PNP, and analyzed, as in (*B*). *D*, the p97-binding site in the C-terminal half of VCIP135. p97-immobilized beads (p97-beads; p97, 0.75 μg) were incubated with either the C-terminal half of VCIP135 or its fragments [(744-1221), 0.60 μg; (744-999), 0.33 μg; (903-1053), 0.50 μg; (1054-1221), 0.40 μg] in the presence of 1 mM AMP-PNP, and analyzed, as in (*B*). *E*, VCIP135 has two distinct p97-binding sites, (1-390) and (903-1053).

As p97 is an ATPase, we first analyzed the effects of nucleotides on the binding between p97 and VCIP135. Figure 1*A* shows that the addition of AMP-PNP, a nonhydrolyzable ATP analogue, increased the binding of VCIP135 to p97 (lane 4; Fig. S1*A*), suggesting that ATP binding enhances the association between p97 and VCIP135. We hence performed the following p97-VCIP135 binding experiments in the presence of AMP-PNP.

VCIP135 was divided into two fragments, VCIP135(1-743) and VCIP135(744-1221), and their binding to p97 was investigated (Figs. 1*B* and S1*B*). To avoid steric hindrance effects of the VCIP135 fragments, p97 was biotinylated and immobilized onto streptavidin-beads, rather than the VCIP135 fragments. Surprisingly, both VCIP135 fragments bound to p97, and the binding efficiency of the N-terminal half was higher than that of the C-terminal half.

We then performed mapping of the p97-binding sites in these VCIP135 fragments. Figure 1*C* shows the results of analysis of the p97-binding site within the N-terminal half of VCIP135. VCIP135(1-390) bound to p97, whereas VCIP135(361-743) did not. Regarding the p97-binding sites in its C-terminal half, only VCIP135(903-1053) bound to p97, but the other fragments did not (Fig. 1*D*). These results show that two distinct p97-binding sites exist in VCIP135, each within fragments (1-390) and (903-1053), as summarized in Figure 1*E*.

**Figure 2:**
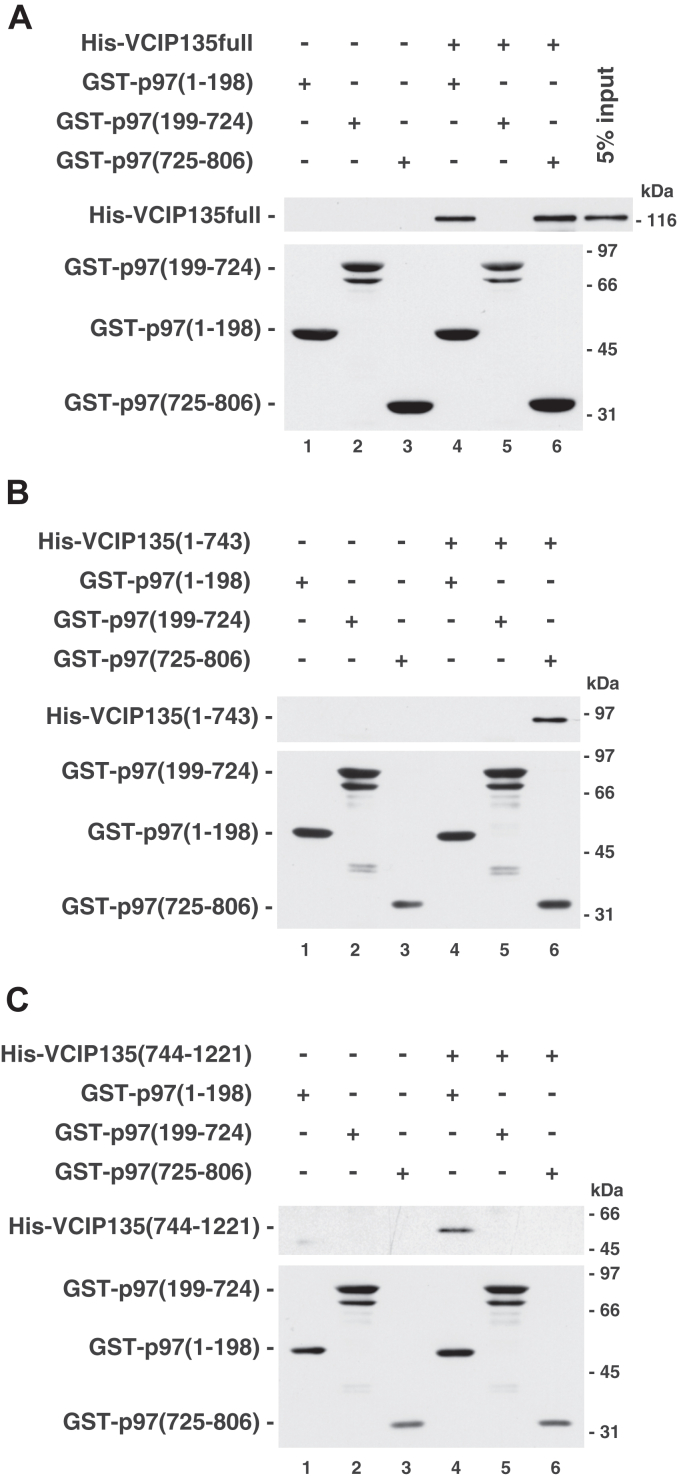
**The N- and C-terminal halves of VCIP135 bind to the C- and N-terminal regions of p97, respectively.***A*, full-length VCIP135 binds to both the N- and C-terminal regions of p97. GST-tagged p97 fragments (4.0 μg) were immobilized on glutathione beads and incubated with full-length VCIP135 (2.0 μg). The bound proteins were analyzed by Western blotting with antibodies to His-tag and GST-tag. *B*, the N-terminal half of VCIP135 binds to the C-terminal region of p97. GST-tagged p97 fragments (4.0 μg) immobilized on glutathione beads were incubated with the N-terminal half of VCIP135 [(1-743), 1.0 μg], and the bound proteins were analyzed, as in (*A*). *C*, the C-terminal half of VCIP135 binds to the N-terminal region of p97. GST-tagged p97 fragments (4.0 μg) immobilized on glutathione beads were incubated with the C-terminal half of VCIP135 [(744-1221), 1.0 μg], and the bound proteins were analyzed, as in (*A*).

We also performed mapping experiments of the VCIP135-binding sites in p97. Interestingly, full-length VCIP135 bound to both the N- and C-terminal regions of p97 (Fig. 2*A*, lanes 4 & 6; Fig. S1*C*). The next question was, which region of p97 interacts with the two distinct binding sites in VCIP135? The results are presented in Figure 2, *B* and *C*. The N- and C-terminal binding sites in VCIP135 interacted with the C- and N-terminal regions of p97, respectively (Fig. 2*B*, lane 6; Fig. 2*C*, lane 4). These results strongly suggest that VCIP135 can associate with p97 *via* two distinct interactions.

### Isolation of nonbinding mutants of the two p97-binding regions in VCIP135

Rough mapping experiments of VCIP135 showed that the regions (1-390) and (903-1053) of VCIP135 are important for its binding to p97. We next aimed to generate mutants of these two p97-binding regions in VCIP135 that do not bind to p97.

**Figure 3:**
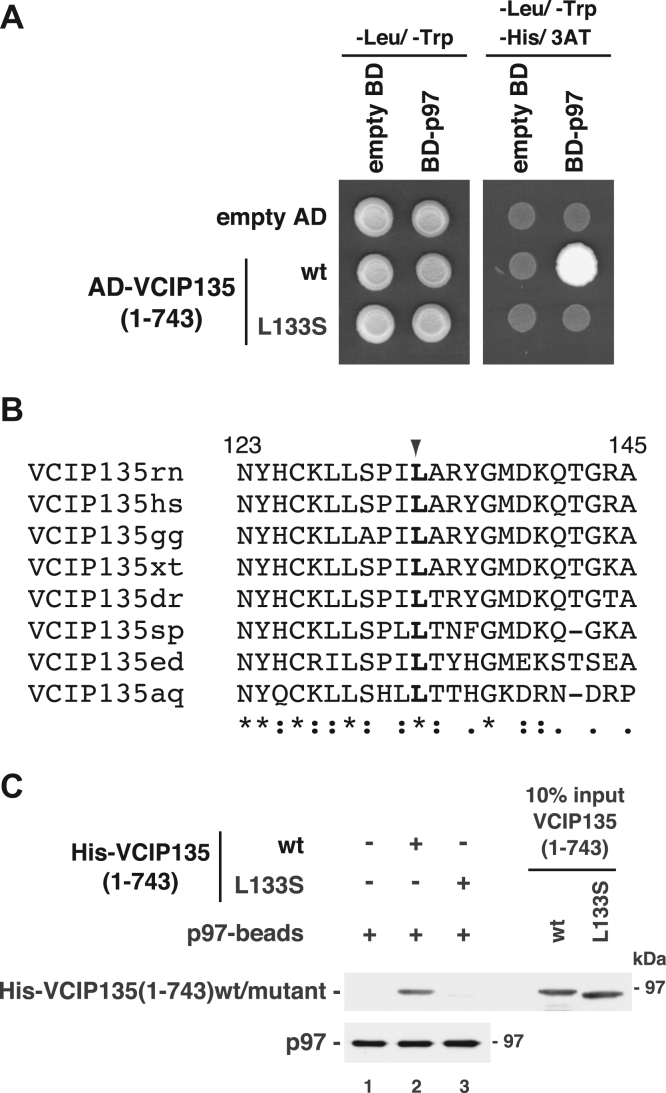
**The L133S mutation in the N-terminal p97-binding site of VCIP135 abolishes its binding to p97.***A*, VCIP135(1-743)(L133S) does not interact with p97 in the yeast two-hybrid experiment. Yeast cells expressing either AD-tagged VCIP135(1-743)wt or its mutant (L133S) and BD-tagged p97 were grown on a histidine-depleted selection plate containing 0.5 mM 3AT (*right panel*). *B*, amino acid sequence alignment of VCIP135 orthologues (rn, *Rattus norvegicus*; hs, *Homo sapiens*; gg, *Gallus gallus*; xt, *Xenopus tropicalis*; dr, *Danio rerio*; sp, *Strongylocentrotus purpuratus*; ed, *Exaiptasia diaphana*; aq, *Amphimedon queenslandica*). Identical residues between the homologues are marked with asterisks. The arrowhead indicates Leu-133 in rat VCIP135. *C*, the N-terminal half of VCIP135 with the L133S mutation has no binding affinity for p97. Either His-tagged VCIP135(1-743)wt or its L133S mutant (2.0 μg) was incubated with p97-immobilized beads (p97-beads; p97, 0.50 μg) in the presence of 1 mM AMP-PNP. The blots were probed with antibodies to p97 and the His-tag.

Regarding the N-terminal p97-binding region, the cDNA of VCIP135(1-390) was amplified by a PCR reaction with a limiting amount of dATP, resulting in the introduction of random mutations. The cDNAs of VCIP135(1-743) containing mutations in theVCIP135(1-390) region were expressed in yeast cells and their p97-binding affinities were tested using a yeast two-hybrid system. We screened approximately 1.6 × 10^3^ colonies and successfully obtained one mutant (L133S) showing low p97-binding affinity (Fig. 3*A*). This Leu-133 is conserved in all VCIP135 orthologues that we found (Fig. 3*B*).

The N-terminal half of VCIP135 with the L133S mutation [VCIP135(1-743) (L133S)] was expressed in *Exaiptasia coli*, collected from the soluble fraction of *E. coli* using Ni-beads, further purified by sucrose-gradient ultracentrifugation and then its biochemical characteristics were analyzed. Figure 3*C* shows the binding of VCIP135(1-743)(L133S) to the p97-immobilized beads. VCIP135(1-743)wt bound to p97 (top panel, lane 2), whereas VCIP135(1-743)(L133S) showed almost no binding to p97 (top panel, lane 3).

**Figure 4:**
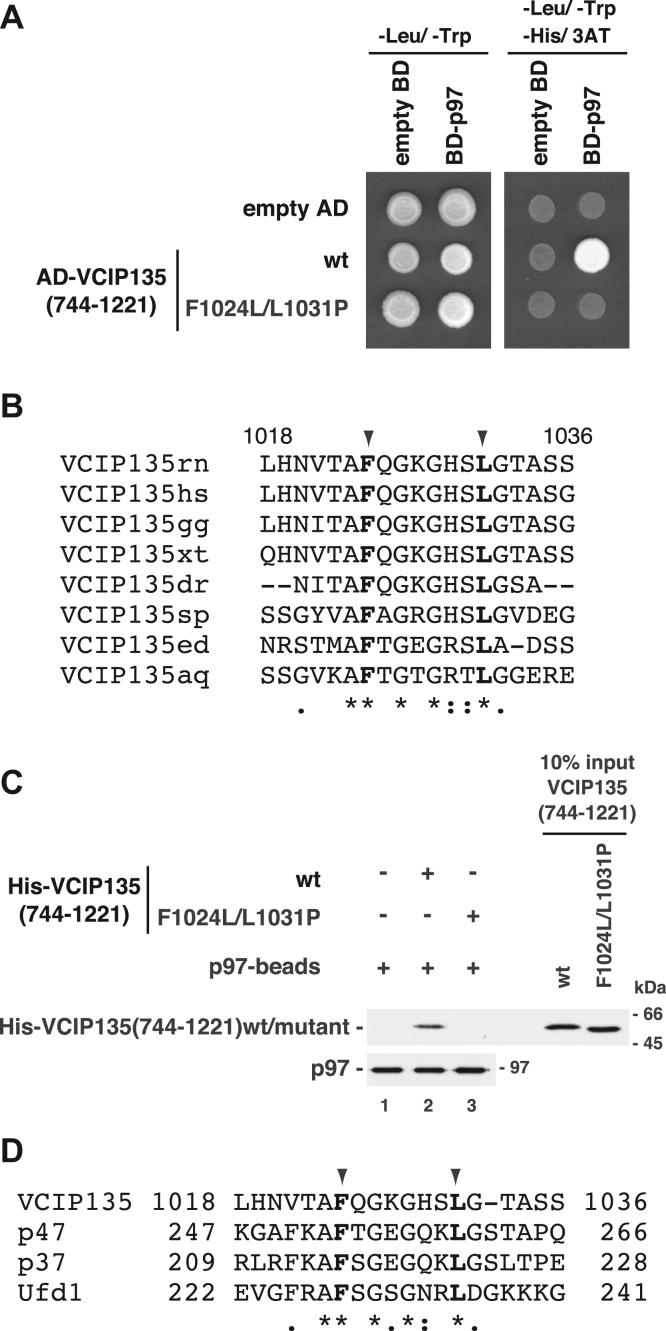
**The F1024L/L1031P mutation in the C-terminal p97-binding site of VCIP135 abolishes its binding to p97.***A*, VCIP135(744-1221) (F1024L, L1031P) does not interact with p97 in the yeast two-hybrid experiment. Yeast cells expressing either AD-tagged VCIP135(744-1221)wt or its mutant (F1024L/L1031P) and BD-tagged p97 were grown on a histidine-depleted selection plate containing 0.5 mM 3AT (*right panel*). *B*, amino acid sequence alignment of VCIP135 orthologues (rn, *Rattus norvegicus*: hs, *Homo sapiens*; gg, *Gallus gallus*; xt, *Xenopus tropicali*s; dr, *Danio rerio*; sp, *Strongylocentrotus purpuratus*; ed, *Exaiptasia diaphana*; aq, *Amphimedon queenslandica*). Identical residues between the homologues are marked with *asterisks*. The *arrowheads* indicate Phe-1024 and Leu-1031 in rat VCIP135. *C*, the C-terminal half of VCIP135 with the F1024L/L1031P mutation shows no binding affinity for p97. Either His-tagged VCIP135(744-1221)wt or its F1024L/L1031P mutant (1.0 μg) was incubated with p97-immobilized beads (p97-beads; p97, 0.50 μg) in the presence of 1 mM AMP-PNP. The blots were probed with antibodies to p97 and the His-tag. *D*, amino acid sequence alignment of VCIP135, p47, p37 and Ufd1. Identical residues are marked with *asterisks*. The *arrowheads* indicate Phe-1024 and Leu-1031 in VCIP135.

Regarding the C-terminal p97-binding region of VCIP135, random mutations were introduced into the (833-1053) region and p97-binding affinities of VCIP135(744-1221) containing the mutated (833-1053) region were analyzed by yeast two-hybrid screening (approximately 1.7 × 10^3^ colonies). As presented in Figure 4*A*, we established the mutant (F1024L/L1031P), which showed low binding affinity to p97 in yeast cells. The amino acids Phe-1024 and Leu-1031 are conserved in all VCIP135 orthologues (Fig. 4*B*). The C-terminal half of recombinant VCIP135 with the F1024L/L1031P mutation was expressed in the soluble fraction of *E. coli*, collected with Ni-beads and further purified by sucrose-gradient ultracentrifugation. Using this mutated VCIP135 fragment, we biochemically confirmed that this mutation abolishes the binding of the C-terminal half of VCIP135 to p97 (Fig. 4*C*, lane 3).

As shown in Figure 4*D*, sequence alignment of VCIP135, p47, p37 and Ufd1 demonstrated the existence of a short homologous stretch, and that Phe-1024 and Leu-1031 in VCIP135 are conserved in p47, p37 and Ufd1.

### Full-length VCIP135 associates with p97 through their two distinct interactions

**Figure 5:**
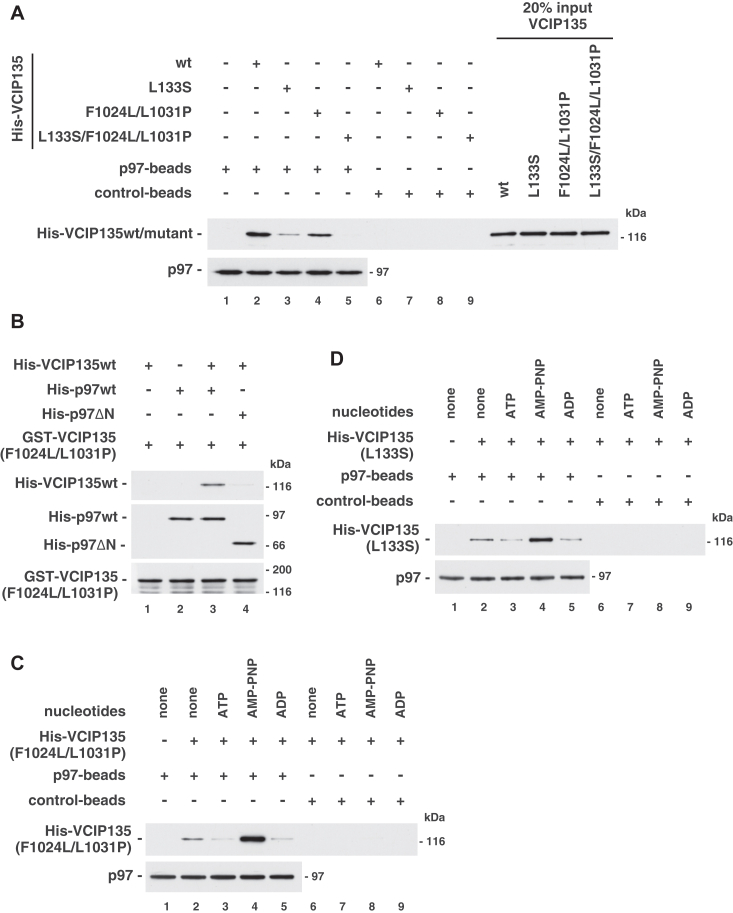
**Full-length VCIP135 associates with p97 through their two distinct interactions.***A*, full-length VCIP135 binds to p97 using either its N- or C-terminal binding site. Either His-tagged full-length VCIP135wt or its mutant (1.0 μg) was incubated with p97-immobilized beads (p97-beads; p97, 0.40 μg) in the presence of 1 mM AMP-PNP. The blots were probed with antibodies to p97 and His-tag. *B*, two full-length VCIP135 molecules can bind to one p97 molecule at its N- and C-terminal regions. GST-tagged VCIP135(F1024L/L1031P) (0.90 μg) was incubated with either His-tagged p97wt (1.2 μg) or p97 (199-806) (p97ΔN, 2.0 μg), and isolated on glutathione beads. The resulting beads were then incubated with His-tagged VCIP135wt (1.0 μg). All reactions were carried out in the presence of 1 mM AMP-PNP. The blots were probed with antibodies to GST-tag and His-tag. *C*, nucleotide dependency of the binding of the N-terminal binding site in VCIP135 to p97. p97-mmobilized beads (p97-beads; p97, 0.40 μg) were incubated with His-tagged VCIP135 (F1024L/L1031P) (1.0 μg) in the presence of the indicated nucleotide (1 mM). The blots were probed with antibodies to p97 and His-tag. *D*, nucleotide dependency of the binding of the C-terminal binding site in VCIP135 to p97. p97-immobilized beads (p97-beads; p97, 0.50 μg) were incubated with His-tagged VCIP135 (L133S) (1.0 μg) in the presence of the indicated nucleotide (1 mM), and analyzed as in (*C*).

To confirm the results from the binding experiments with the VCIP135 fragments, we next investigated whether full-length VCIP135 utilizes these two interactions for its association with p97. We prepared recombinant full-length VCIP135 mutant proteins, in which either or both of the N- and C-terminal regions lacked the affinity for p97 and analyzed their binding to p97. These VCIP135 mutant proteins were further purified by sucrose-gradient ultracentrifugation to remove misfolded and aggregated proteins. The VCIP135 mutant (L133S/F1024L/L1031P), in which both p97-binding sites were mutated, is no longer bound to p97 (Fig. 5*A*, lane 5). The VCIP135 mutants (L133S) and (F1024L/L1031P), in which only one of the two p97-binding sites was intact, still bound to p97, although their binding affinities were decreased compared with that of VCIP135wt (Fig. 5*A*, lanes 2–4; Fig. S1*D*). The decrease in binding affinity was much larger in VCIP135(L133S) than in VCIP135 (F1024L/L1031P) (Fig. 5*A*, lanes 3 & 4; Fig. S1*D*), which is consistent with the observation that the N-terminal half of VCIP135 showed a higher binding affinity for p97 than the C-terminal half (Fig. 1*B* and S1*B*).

Considering that each of these two binding interactions is sufficient to form a complex, we expected that one molecule each of VCIP135 binds to the N- and C-terminal regions of a p97 molecule. To confirm this, we performed the following binding experiments (Fig. 5*B*). Briefly, GST-VCIP135(F1024L/L1031P), which binds to the C-terminus of p97 but not to its N-terminus, was incubated with p97, and immobilized onto GSH-beads. The resulting beads were incubated with His-VCIP135wt. As shown in Figure 5*B*, the complex of VCIP135(F1024L/L1031P) and p97 was able to associate with another VCIP135 molecule (lane 3). When the p97 mutant (199–-806; p97ΔN) was used instead of p97wt, the association of another VCIP135 molecule was not observed (lane 4). These results indicate that p97 can associate with two VCIP135 molecules using the two binding interactions. In other words, these results strongly support the fact that each of the two distinct binding interactions between VCIP135 and p97 is sufficient for VCIP135-p97 complex formation.

We also investigated the nucleotide dependency of the two interactions between VCIP135 and p97 using these full-length VCIP135 mutants. As shown in Figure 5, *C* and *D*, the binding of both VCIP135 mutants, in which only one of the two p97-binding sites was intact, were increased in the presence of AMP-PNP (lane 4). These results indicate that the binding of ATP to p97 enhances both of these interactions between VCIP135 and p97; that is, these two interactions in the complex may be synchronously controlled by the nucleotide state of p97. This nucleotide-dependent synchrony of the two interactions, which is consistent with the nucleotide-dependent association between VCIP135wt and p97 (Fig. 1*A*), implies their functional significance.

### The two interactions between VCIP135 and p97 are required for Golgi and ER biogenesis *in vivo*

**Figure 6:**
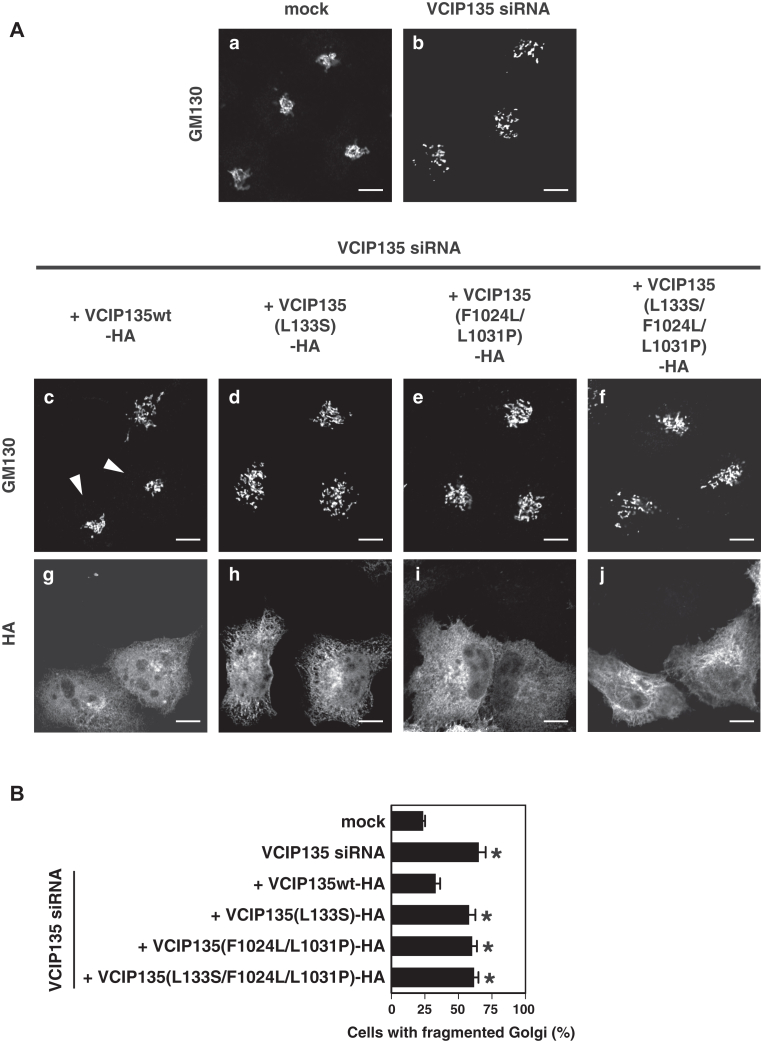
**The two binding interactions between VCIP135 and p97 are required for Golgi biogenesis *in vivo*.***A*, rescue experiments in VCIP135 siRNA-treated cells. HeLa cells were either mock transfected with water or transfected with siRNA duplexes specific to human VCIP135, and cultured for 46 h. In some groups, the indicated HA-tagged VCIP135wt/mutants, which were insensitive to VCIP135 siRNA, were expressed 18 h after the treatment of the cells with VCIP135 siRNA, and the cells were further cultured for 28 h. Levels of endogenous and exogenous VCIP135 in the cultured cells are presented in Fig. S2*A*. The resulting cells were fixed and stained with a polyclonal antibody to GM130 (*panels a–f*) and a monoclonal antibody to HA (*panels g–j*). The treatment of cells with VCIP135 siRNA caused Golgi fragmentation (*panel b*). In VCIP135 siRNA-treated cells, the Golgi morphology was rescued by the expression of VCIP135wt (*panel c*, *white arrowheads*), but not by the expression of VCIP135 mutants in which either or both of the N- and C-terminal p97-binding sites were mutated (*panels d*–*f*). Scale bar = 5 μm. *B*, results of the quantification of (*A*). Results are expressed as the mean ± SD of six sets of independent experiments, with 100 cells counted in each group of each set. Asterisks indicate a significant difference at *p* < 0.01 compared with mock-treated cells (Bonferroni method).

We previously reported that both VCIP135 and p97, which form a complex in the cytosol, bind to Golgi and ER membranes, and function in Golgi and ER biogenesis (6, 8). However, it remains unclear whether the interactions between VCIP135 and p97 are required for their biogenesis. We hence aimed to clarify the *in vivo* roles of the two binding interactions between VCIP135 and p97. We first performed siRNA experiments by introducing a human VCIP135 siRNA into HeLa cells, and observed the morphology of the Golgi. Western blotting showed that the VCIP135 siRNA duplex significantly decreased the intracellular level of VCIP135 (Fig. S2*A*). In VCIP135-depleted cells, the Golgi was fragmented and dispersed (Fig. 6*A*, panel b). The treatment of cells with VCIP135 siRNA significantly increased the ratio of cells with fragmented Golgi (Fig. 6*B*, second bar from the top).

We next performed rescue experiments in VCIP135-depleted cells. HA-tagged rat VCIP135 (VCIP135-HA), which was insensitive to human VCIP135 siRNA, was expressed in human VCIP135 siRNA-treated HeLa cells. The expression of VCIP135wt-HA rescued the Golgi structures in VCIP135-depleted cells (Fig. 6*A*, panels c & g, white arrowheads; Fig. 6*B*, third bar from the top). On the other hand, the expression of VCIP135 mutants, in which either or both N- and C-terminal p97-binding sites lacked affinity for p97, did not rescue the Golgi morphology in VCIP135-depleted cells (Fig. 6*A*, panels d-f & h-j; Fig. 6*B*, fourth, fifth and sixth bars). These *in vivo* results strongly suggest that both of the two interactions between VCIP135 and p97 are required for Golgi biogenesis.

**Figure 7:**
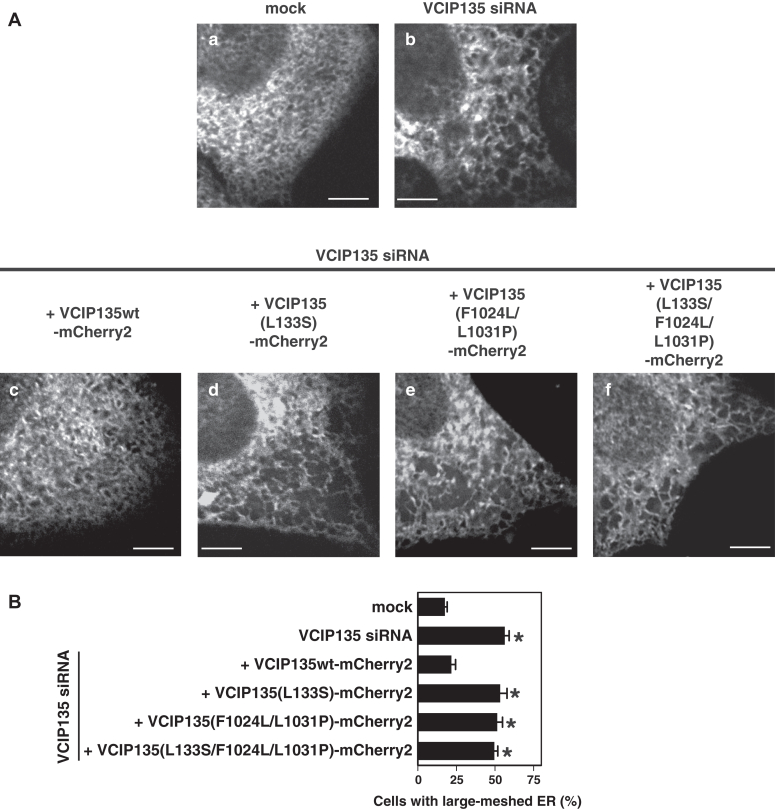
**The two binding interactions between VCIP135 and p97 are necessary for ER biogenesis *in vivo*.***A*, effects of the expression of VCIP135 mutants on ER structure in VCIP135 siRNA-treated cells. The HeLa cells stably expressing GFP-tagged HSP47 were used, and their ER structures were observed in living cells. The HeLa cells were either mock transfected with water or transfected with siRNA duplexes specific to human VCIP135 and cultured for 44 h. In some groups, the indicated mCherry2-fused VCIP135wt/mutants, which were insensitive to VCIP135 siRNA, were expressed 16 h after the treatment of cells with VCIP135 siRNA, and the cells were further cultured for 28 h. Levels of endogenous and exogenous VCIP135 in the cultured cells are presented in Fig. S2*B*. Cells expressing mCherry2-fused VCIP135 were used to obtain images. Confocal images of cultured cells were obtained at the height half way between the base and equatorial plane of the nucleus. In VCIP135 siRNA-treated cells, the fine-meshed network of the ER was found to be disrupted in numerous areas, resulting in the appearance of large holes (*panel b*). The ER morphology was rescued by the expression of VCIP135wt (*panel c*), but not by the expression of VCIP135 mutants in which either or both of the N- and C-terminal p97-binding sites were mutated (panels d-f). Scale bar = 5 μm. *B*, results of the quantification of (*A*). Results are expressed as the mean ± SD of five sets of independent experiments, with 100 cells counted in each group of each set. *Asterisks* indicate a significant difference at *p* < 0.01 compared with mock-treated cells (Bonferroni method).

We also previously reported that both VCIP135 and p97 bind to ER membranes and function in ER network formation (8, 9). We therefore investigated the roles of the interactions between VCIP135 and p97 in ER network formation in living cells. As ER structures are easily damaged and lost by fixation, we used a stable cell line expressing GFP-tagged HSP47, an ER protein, for the *in vivo* experiments, and observed ER structures in living cells without fixation by confocal microscopy. In VCIP135 siRNA-treated cells, the fine-meshed network of the ER was found to be disrupted in numerous areas, resulting in the appearance of large holes (Fig. 7*A*, panel b). The treatment of cells with VCIP135 siRNA significantly increased the ratio of cells with a large-meshed ER network (Fig. 7*B*, second bar from the top). The ER morphology in VCIP135-depleted cells was rescued by the expression of VCIP135wt (Fig. 7*A*, panel c; Fig. 7*B*, third bar from the top), but not by the expression of VCIP135 mutants in which either or both of the N- and C-terminal p97-binding sites were mutated (Fig. 7*A*, panels d-f; Fig. 7*B*, fourth, fifth and sixth bars).

In summary, our results demonstrate that VCIP135 functions in Golgi biogenesis and ER network formation by forming a complex with p97; particularly, both of the binding interactions in the complex are essential for VCIP135 function.

## Discussion

We previously identified two p97ATPase-mediated membrane fusion pathways, namely, the p97/p47 pathway and the p97/p37 pathway (3, 4). Both of these pathways utilize many p97-binding cofactors; that is, p47, p37, and VCIP135 as direct p97-binding cofactors, and WAC as an indirect p97-binding cofactors (3, 4, 8, 9). We recently identified one more direct p97-binding cofactor, FTCD, in p97/p47-mediated Golgi membrane fusion (6). This led to the important question as to how p97 selects and changes its cofactors during the membrane fusion process. At present, we do not have an answer to this. Strictly speaking, only a few of the cofactors have actually been shown to function by forming a complex with p97 in membrane fusion (6, 13), and the others are still only “potential” p97 binding partners. VCIP135 is one such “potential” p97 binding partner in p97-mediated membrane fusion. Furthermore, VCIP135 has both ubiquitin-dependent and -independent functions in p97-mediated membrane fusion (9), and has recently been reported to function as a deubiquitinase without requiring the assistance of p97 (12). To clarify whether VCIP135 functions in p97 membrane fusion by forming a complex with p97, in the present study, we first aimed to identify its p97-binding site. Surprisingly, VCIP135 has two distinct p97-binding sites in its N- and C-terminal regions. This is inconsistent with our previous report that VCIP135 has only one p97-binding site at its C-terminal region (8). In our previous study, His-tagged VCIP135(1-743), which was immobilized onto protein-G beads using anti-His tag antibodies, did not bind to p97. One possible explanation is that the anti-His antibodies used for the immobilization might have prevented p97 from approaching the p97-binding site in VCIP135(1-743). In the present study, to avoid such steric hindrance, p97 was biotinylated and immobilized onto streptavidin-beads, followed by its incubation with VCIP135 fragments.

The existence of these two binding interactions between p97 and VCIP135 has demonstrated several interesting points. First, the N- and C-terminal binding sites in VCIP135 interacted with the C- and N-terminal regions of p97, respectively (Fig. 2, *B* and *C*). To the best of our knowledge, VCIP135 is the first protein that has been identified to interact with both the N-and C-terminal regions of p97. Second, the two p97-binding sites of VCIP135 exist far away from each other, at its N- and C-terminal regions. Although several p97-binding proteins, such as p47 and p37, are known to have two binding sites for p97, their sites are localized close together (13, 14). The existence of a long flexible linker between the two p97-binding sites in VCIP135 is thought to enable one VCIP135 molecule to associate with both of the N- and C-terminal regions of one p97 molecule. Third, these two binding interactions in the complex are synchronously controlled by the nucleotide state of p97 (Fig. 5, *C* and *D*), which leads to the nucleotide-dependent association between full-length VCIP135 and p97 (Fig. 1*A*). Fourth, the C-terminal p97-binding site of VCIP135 has a short homologous stretch, and Phe-1024 and Leu-1031 in VCIP135 is conserved in p47, p37 and Ufd1 (Fig. 4*D*). It was reported that p47 (F253S), p37 (F215S) and Ufd1(F228S), which correspond to the Phe-1024 mutant of VCIP135, do not have binding affinity for p97 (13), and the short homologous stretch among these proteins known as the SHP motif is a p97/Cdc48p-binding motif (14, 15). As the C-terminal half of VCIP135 with the F1024L/L1031P mutation does not bind to p97 (Fig. 4*C*), the C-terminal p97-binding site in VCIP135 is also thought to contain a SHP motif. Finally, each of the two binding interactions between VCIP135 and p97 is sufficient for their complex formation. VCIP135 mutants with only one of the two p97 binding interactions still formed a complex with p97 (Fig. 5*A*). This is also supported by the existence of the VCIP135-p97-VCIP135 complex (Fig. 5*B*), even though it is unclear whether this complex exists stably in living cells.

It is known that the phosphorylation of VCIP135 during mitosis disrupts its association with p97 (16, 17). We previously showed that VCIP135 is phosphorylated on Thr-760 and Ser-767 by Cdc2 during mitosis and that the phosphorylation of VCIP135 decreases its binding affinity for p97 and inhibits p97-mediated Golgi membrane fusion during mitosis (16). In the present study, Thr-760 and Ser-767 were not identified as p97-binding sites. One possible explanation is that this phosphorylation might cause a conformational change in the linker region between the two binding sites in VCIP135, resulting in the dissociation of p97 from each or both of the two binding sites in VCIP135. It was also reported that VCIP135 is mitotically phosphorylated on Ser-130, which weakens its binding to p97 (17). The phosphorylation of Ser-130 might disrupt the binding of p97 to the N-terminal binding site in VCIP135.

In the present study, we clarified that VCIP135 and p97 form a complex through two distinct interactions and that either one of the interactions is sufficient for this complex formation. Therefore, the next question was whether these binding interactions are necessary for p97-mediated membrane fusion. Although both p97 and VCIP135 are required for p97-mediated membrane fusion, it was unclear whether they function together by forming a complex. We hence investigated the functional significances of these two binding interactions in p97-mediated Golgi and ER biogenesis (Figs. 6 and 7). The depletion of VCIP135 caused fragmented and dispersed Golgi and a large-meshed ER network in cultured cells, and these morphological changes were rescued by the expression of VCIP135wt, but not by the expression of the VCIP135 mutant in which neither of the two p97-binding sites was intact. This indicates that the association between p97 and VCIP135 is necessary for Golgi and ER biogenesis. Interestingly, these morphological changes were not rescued by the expression of VCIP135 mutants with only one of the two p97 binding sites, indicating that these two distinct interactions between VCIP135 and p97 are essential for Golgi and ER biogenesis. On the other hand, we previously showed that VCIP135 has two distinct functions in p97-mediated membrane fusion, namely, ubiquitin-dependent and ubiquitin-independent functions (9). VCIP135 was reported to function as a deubiquitinating enzyme without the assistance of p97; that is, in the activation of SPRTN, a specialized DNA-dependent metalloprotease, VCIP135 deubiquitinated SPRTN in the absence of p97 (12). We also showed that the binding of VCIP135 to p97 does not change its deubiquitinating activity (9). In addition, ER biogenesis, which was reported to require VCIP135 but not its deubiquitinating activity (9), has been shown to require its binding to p97 (Fig. 7). Taking these facts altogether, it is plausible that these binding interactions of VCIP135 with p97 may be required for the ubiquitin-independent functions of VCIP135, but not for its ubiquitin-dependent function.

Finally, we will discuss the functional significance of these two binding interactions in the VCIP135-p97 complex. VCIP135 can bind to both the N- and C-terminal regions of p97. This suggests that VCIP135 can compete simultaneously with two other distinct cofactors that bind to the N- and C-terminal regions of p97. We recently reported that p97 forms a big tethering complex, FTCD-p97-p47-FTCD, with p47 and FTCD in p97/p47-mediated Golgi membrane fusion. In this tethering complex, p97 binds to p47 and FTCD at its N- and C-terminal regions, respectively (6). VCIP135 might compete simultaneously with p47 and FTCD for binding to the N- and C-terminal regions of p97, respectively. We also showed previously that VCIP135 binds to the p97-p47 complex, which leads to the dissociation of the complex as a result of ATP hydrolysis by p97 (8). In addition, it has been shown that when p47 and p97 dissociate from each other, the binding affinity between p47 and FTCD weakens (6). Considering these facts altogether, there is a possibility that VCIP135 can dissociate the FTCD-p97-p47-FTCD tethering complex *via* p97-mediated ATP hydrolysis. The tethering complex is generally thought to be dissociated and then reused after membrane fusion, and hence VCIP135 might be a candidate recycling factor of the tethering complex.

Another important point is that VCIP135 can associate with p97 by binding to either its N- or C-terminal region. As all p97 cofactors except for VCIP135 bind to either the N- or C-terminal region of p97, VCIP135 is expected to associate, albeit temporarily, with the other region of p97 to form an unstable triplet complex, that is, other cofactor-p97-VCIP135 (Fig. S3). Then, as VCIP135 might be able to interact more stably with p97 *via* two binding interactions, the other cofactor may dissociate from p97. This means that VCIP135 can work as a dissociation factor for p97-containing complexes. In particular, as p47, p37, Ufd1, and VCIP135 share the same p97-binding motif (SHP motif), it is reasonable that VCIP135 may induce the dissociation of complexes of p97 with p47, p37, and Ufd1. In fact, we previously showed that VCIP135 functions as a dissociation factor for the p97-p47 complex, with the assistance of p97-mediated ATP hydrolysis (8). p97 utilizes several distinct cofactors, such as p47, p37, FTCD, and VCIP135, for p97-mediated membrane fusion, and how it changes its cofactors during this process is an important issue, in which VCIP135 may be a key player.

## Experimental procedures

### Proteins, constructs, antibodies, and siRNAs

Recombinant p97, VCIP135, and their fragments were prepared from the soluble fractions of *E.coli* using tag-affinity beads and further purified by sucrose-gradient ultracentrifugation, as reported previously (3, 8, 9). For the production of mutated proteins, mutations were directly introduced into their cDNA in either the pTrcHis or pGEX-6P-1 vector by PCR reactions, using the QuikChange mutagenesis kit (Stratagene). All clones were verified by DNA sequencing.

For the expression of HA-tagged VCIP135 and its mutants in cultured cells, their rat cDNAs were subcloned into the pCG-C-BL vector. For the expression of mCherry2-fused proteins in cultured cells, their rat cDNAs were subcloned into the mCherry2-N1 vector.

Polyclonal antibodies to VCIP135, p97 and GM130 were prepared as described previously (5, 8, 9). Monoclonal antibodies to GST and α-tubulin were purchased from Sigma; monoclonal antibodies to p97, His-tag and HA-tag were from Progen, Proteintech and MBL, respectively.

Human VCIP135 was targeted using the following siRNA duplex: 5′-GGCAUGCCUUAAGAGAGAAUCUUAA -3′ (VCIP135 siRNA).

### Biochemical experiments

Endogenous p97 was purified from rat liver cytosol (18), biotinylated using EZ-Link Sulfo-NHS-LC-biotin (Pierce) and bound to streptavidin-beads (Sigma-Aldrich). GST-tagged p97, VCIP135 and their fragments were bound to GSH-beads (Cytiva). The resulting protein-bound beads were used for binding experiments in buffer (20 mM HEPES, 100 mM KCl, 1 mM MgCl2, 1 mM DTT, 0.5% CHAPS, 10% glycerol, pH 7.4). For the binding experiments using full-length p97, buffer containing 1 mM AMP-PNP was used, unless otherwise stated.

For the binding experiments, tagged proteins were used and Western blotting was performed using anti-tag antibodies for their detection to estimate and compare the amounts of binding proteins with different molecular weights.

### Isolation of VCIP135 mutants lacking binding affinity to p97

VCIP135 mutants were generated and screened as previously described (13, 19). For analysis of the N-terminal p97-binding site, the cDNAs of VCIP135(1-194) and VCIP135(195-390) were amplified by a PCR reaction in which dATP was limited to 10% of the other dNTPs, to generate PCR products with mutations randomly distributed throughout VCIP135(1-390). The cDNAs of VCIP135(1-743), in which mutations were randomly introduced in its (1-390) region, were generated using its PCR products, and then subcloned into the pACT2 vector. In the case of the C-terminal p97-binding site, the cDNA of VCIP135(833-1053) was amplified by a PCR reaction with a limited amount of dATP, and used to generate VCIP135(744-1221) in the pACT2 vector. The ORF of p97 was also subcloned into the pGBKT7 vector. The resulting pACT2 and pGBKT7 plasmids were cotransformed into yeast strain AH109. Yeast cells expressing either activation domain (AD)-tagged VCIP135(1-743) mutants or VCIP135(744-1221) mutants together with DNA-binding domain (BD)-tagged p97 were screened on histidine-depleted selection plates containing 0.5 mM 3-amino-1,2,4-triazole (3AT).

### Analysis of Golgi morphology in cultured cells

HeLa cells were either mock transfected with water or transfected with VCIP135 siRNA duplex (30 nM) using TransIT-X2 (Mirus). After incubation for 46 h, the cells were fixed and used to observe the Golgi structures. For the rescue experiments, the mammalian expression construct (0.44 mg/l) of the HA-tagged rat VCIP135wt/mutant, which is insensitive to human VCIP135 siRNA, was transfected using TransIT-X2, 18 h after the treatment of cells with VCIP135 siRNA and the cells were further cultured for 28 h.

For the observation of Golgi structures, cells grown on coverslips were fixed with 3% PFA/PBS for 6 min, permeabilized with 0.1% T × 100 for 5 min, and stained with polyclonal antibodies to GM130 and monoclonal antibodies to HA-tag. The stained cells were observed using a confocal microscope (Zeiss LSM700) equipped with a 63× objective lens (Plan-Apochromat, NA 1.40).

### Analysis of ER morphology in cultured cells

To observe ER structures, a stable HeLa cell line expressing GFP-tagged HSP47, an ER protein, was used (8). ER structures were observed and their images were taken in living cells without fixation, using a spinning disk confocal microscope (CSU-X1, YOKOGAWA; iXon+ CCD camera, Andor) equipped with a 60× objective lens (UPlanApo, NA 1.50, Olympus) and a stage top incubator (INUG2F, Tokai Hit).

The cells were either mock transfected with water or transfected with the VCIP135 siRNA duplex (30 nM) using Lipofectamine RNAimax (Thermo Fisher Scientific), and incubated for 44 h. For the rescue experiments, the cells were transfected with the mammalian expression construct (0.44 mg/l) of mCherry2-fused rat VCIP135wt/mutant using Lipofectamine 3000 (Thermo Fisher Scientific), 16 h after VCIP135 siRNA treatment and further cultured for 28 h. Cells expressing mCherry2-fused VCIP135 were used to obtain images. Confocal images of cultured cells were obtained at the height half way between the base and equatorial plane of the nucleus.

## Data availability

All data are contained within the manuscript.

## Supporting information

This article contains Supporting information.

## Conflicts of interest

The authors declare that they have no conflicts of interest with the contents of this article.
