# Supplementary material for: VCIP135 associates with both the N- and C-terminal regions of p97 ATPase
Source: J Biol Chem. 2023 Dec 10;300(1):105540. doi: 10.1016/j.jbc.2023.105540 (PMC10805704; doi:10.1016/j.jbc.2023.105540)
Supplement: Supporting Figures S1–S3 [file mmc1.pdf]

VCIP135 associates with both the N- and C-terminal regions of p97 ATPase

Suzune Nakayama and Hisao Kondo

Department of Molecular Cell Biology, Graduate School of Medical Sciences, Kyushu University, Fukuoka 812-8582, Japan

SUPPORTING INFORMATION: FIGURE LEGENDS

Figure S-1. Quantification of results

(A) Quantification of the results of Figure 1A.

(B) Quantification of the results of Figure 1B.

(C) Quantification of the results of Figure 2A.

(D) Quantification of the results of Figure 5A.

Figure S-2. Levels of endogenous and exogenous VCIP135 in cultured cells

(A) Levels of endogenous and exogenous VCIP135 in the cultured cells presented in Figure 6. Cells were analyzed by Western blotting with antibodies to VCIP135 and α-tubulin.

(B) Levels of endogenous and exogenous VCIP135 in the cultured cells presented in Figure 7. Cells were analyzed by Western blotting with antibodies to VCIP135 and α-tubulin.

Figure S-3. The working model: VCIP135 acts as a dissociation factor for p97-containing complexes

(1) Factor X binds to the N-terminal region of p97 to form the Factor X-p97 complex. (2) VCIP135 is expected to associate, albeit temporarily, with the C-terminal region of p97 to form an unstable triplet complex, i.e., Factor X-p97-VCIP135. (3) Then, as VCIP135 can interact more stably with p97 via two binding interactions, Factor X dissociates from p97. (4) Next, instead of Factor X, Factor Y has a chance to form the Factor Y-p97-VCIP135 complex. (5) The resulting triplet complex will be dissociated through p97 ATP hydrolysis to form the Factor Y-p97 complex.

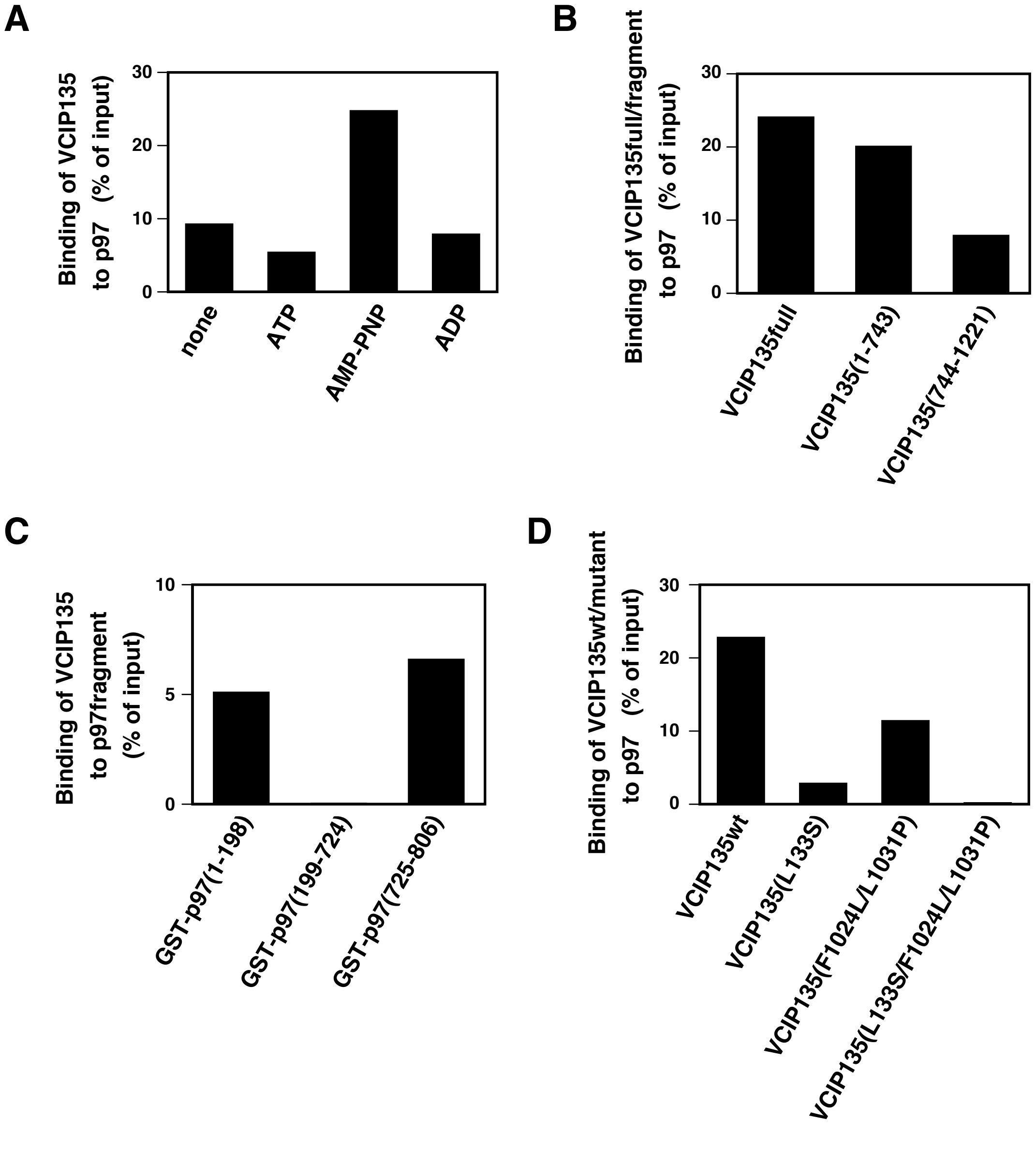
A
Binding of VCIP135 to p97 (% of input)
30
20
10
0
none
ATP
AMP-PNP
ADP
B
Binding of VCIP135full/fragment to p97 (% of input)
30
20
10
0
VCIP135full
VCIP135(1-743)
VCIP135(744-1221)
C
Binding of VCIP135 to p97fragment (% of input)
10
5
0
GST-p97(1-198)
GST-p97(199-724)
GST-p97(725-806)
D
Binding of VCIP135wt/mutant to p97 (% of input)
30
20
10
0
VCIP135wt
VCIP135(L133S)
VCIP135(F1024L/L1031P)
VCIP135(L133S/F1024L/L1031P)

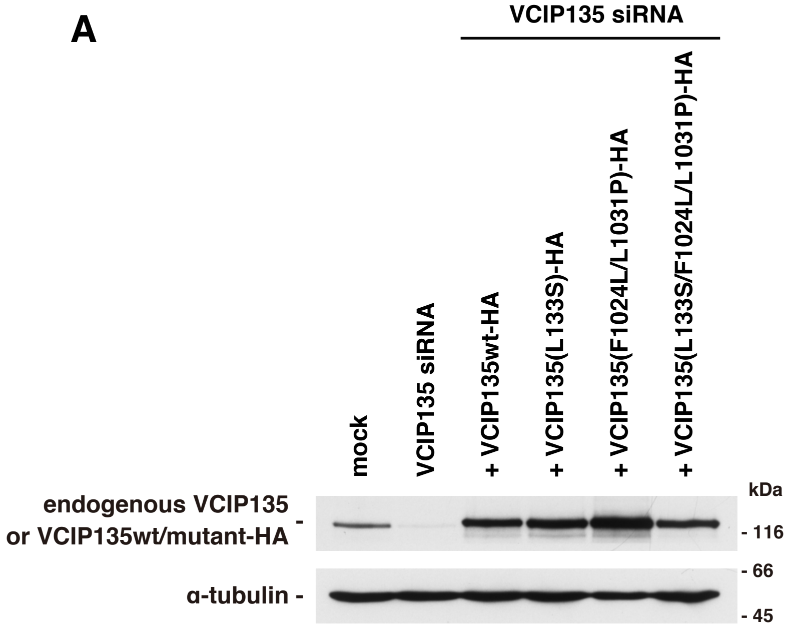
A
VCIP135 siRNA
mock
VCIP135 siRNA
+ VCIP135wt-HA
+ VCIP135(L133S)-HA
+ VCIP135(F1024L/L1031P)-HA
+ VCIP135(L133S/F1024L/L1031P)-HA
kDa
endogenous VCIP135
or VCIP135wt/mutant-HA
- 116
- 66
α-tubulin
- 45

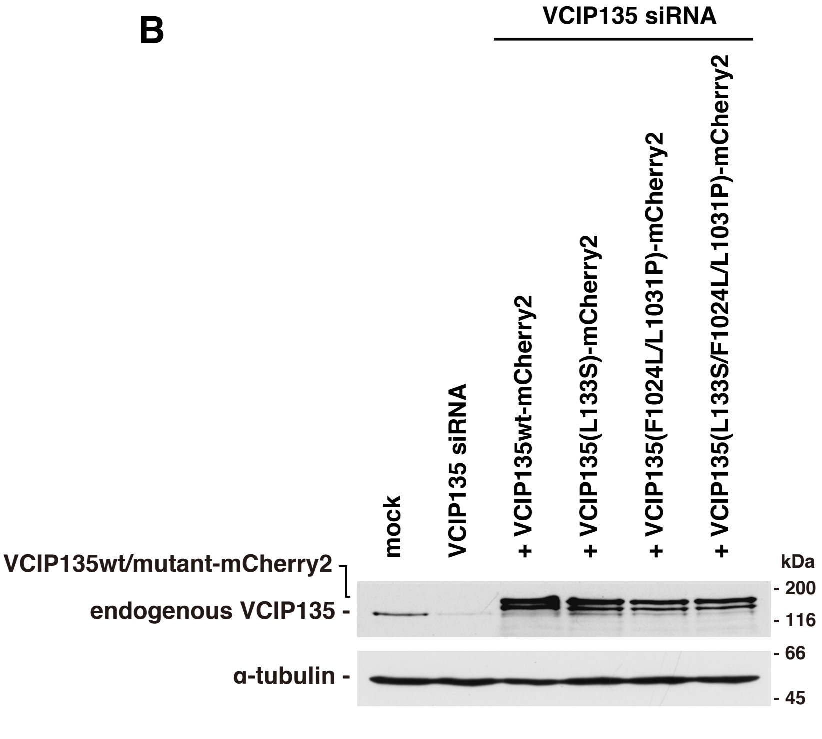
B
VCIP135 siRNA
mock
VCIP135 siRNA
+ VCIP135wt-mCherry2
+ VCIP135(L133S)-mCherry2
+ VCIP135(F1024L/L1031P)-mCherry2
+ VCIP135(L133S/F1024L/L1031P)-mCherry2
kDa
VCIP135wt/mutant-mCherry2
endogenous VCIP135
- 200
- 116
- 66
α-tubulin
- 45

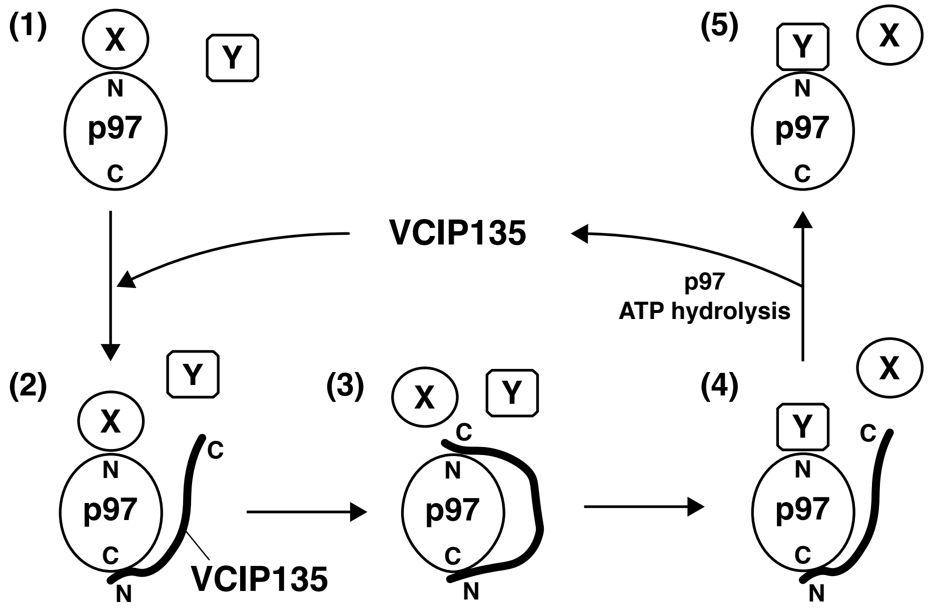
(1)
X
Y
N
p97
C
(5)
Y
X
N
p97
C
VCIP135
p97
ATP hydrolysis
(2)
Y
X
N
p97
C
C
N
VCIP135
(3)
X
Y
C
N
p97
C
N
(4)
X
Y
C
N
p97
C
N
